# Early-life socioeconomic circumstances and the comorbidity of depression and overweight in adolescence and young adulthood: A prospective study

**DOI:** 10.1016/j.ssmph.2023.101494

**Published:** 2023-08-19

**Authors:** Fanny Kilpi, Laura D. Howe

**Affiliations:** a MRC Integrative Epidemiology Unit at the University of Bristol, Bristol, UK; b Population Health Sciences, Bristol Medical School, University of Bristol, Bristol, UK

**Keywords:** Depression, BMI, Comorbidity, Adolescence, Socioeconomic position, Alspac

## Abstract

Depression and overweight both often emerge early in life and have been found to be associated, but few studies examine depression-overweight comorbidity and its social patterning early in the life course. Drawing on data from 4,948 participants of the Avon Longitudinal Study of Parents and Children (ALSPAC) birth cohort from the UK (2,798 female, 2,150 male), we investigated how different aspects of early-life socioeconomic circumstances are associated with depression-overweight comorbidity from adolescence to young adulthood exploring any differences by age and sex. We estimated how parental education, social class and financial difficulties reported in pregnancy were associated with depression and overweight, and their comorbidity at approximately the ages 17 and 24 in males and females. The results from multinomial logistic regression models showed that all three socioeconomic markers were associated with depression-overweight comorbidity and results were similar across age. Lower parental education (relative risk ratio (RRR) and 95% confidence interval (CI) of low education v high education: 3.61 (2.30-5.67) in females and 1.54 (1.14-2.07) in males) and social class (class IV/V v class I: 5.67 (2.48-12.94) in females and 3.11 (0.70-13.91) in males) had strong associations with comorbidity at age 17 relative to having neither depression or overweight. Financial difficulties were also a risk factor in females, with less clear results in males. These findings highlight how early socioeconomic circumstances are linked with the accumulation of mental and physical health problems already in adolescence, which has implications for life-long health inequalities.

## Highlights

• Early socioeconomic circumstances are linked with health in adolescence.
• We find it linked with the comorbidity of depression and overweight in adolescents.
• Results show the social patterning of the early accumulation of health problems.
• Parental education is a stronger factor than class or financial difficulties.

## 1 Introduction

Overweight or obesity and depression in adolescence and young adulthood are important public health concerns as both tend to track into later life (Fergusson et al., 2007; Norris et al., 2019; Singh et al., 2008). Overweight and depression may share risk factors and aetiology (Milaneschi et al., 2019), and studies have also consistently demonstrated a reciprocal relationship between overweight and depression that may begin already at a young age (Davillas et al., 2016; de Wit et al., 2010; Konttinen et al., 2014; Luppino et al., 2010; Mannan et al., 2016; Patalay & Hardman, 2019; Stunkard et al., 2003; Sutaria et al., 2018). The comorbidity of overweight and depression is likely to be one of the earliest common life course manifestations of multimorbidity, with potentially serious and lifelong implications for subsequent chronic diseases and healthcare costs. However, their comorbidity in adolescence and young adulthood has been seldom studied, with research to date focusing on the accumulation of health problems in later life (Khanolkar & Patalay, 2021). Nevertheless, this period is formative in the development of physical and mental health and health-related behaviours, and an important life stage during which influences from the family translate to health.

Family socioeconomic circumstances have been found to be associated with higher adolescent depressive symptoms (Björkenstam et al., 2017; Joinson et al., 2017; Reiss, 2013; Wirback et al., 2014) and body mass index (BMI) (Barriuso et al., 2015; Shrewsbury & Wardle, 2008; Wardle et al., 2006). Disadvantaged circumstances may increase individuals’ exposure to stress and ability to access resources that help cope with stress. Socioeconomic position has also been associated with cross-sectional associations between mental and physical health (Sacker et al., 2009). There may be a ‘vicious cycle’ between stress/depressive symptoms and obesity, partly due to the social stigma linked with obesity (Tomiyama, 2019), which can have an impact on outcomes throughout the life course. Thus if social conditions are linked to the co-occurrence and interaction between depression and overweight, there is a potentially important public health impact of intervening on these social causes. This perspective draws from the syndemics framework, which recognizes that diseases do not occur in isolation, and there can be major public health implications of disease and social environment interactions (Singer et al., 2017).

The socioeconomic position (SEP) of the family in general reflects the life chances available through money, power, knowledge and prestige (Link & Phelan, 1995). However, parental education, social class and financial difficulties reflect access to somewhat different resources and exposure to different patterns of risk factors, which may not be associated with adolescent health exactly the same way (Braveman et al., 2005; Geyer et al., 2006; Shrewsbury & Wardle, 2008; Sobal, 1991). Education, for example, may influence BMI through access to knowledge about healthier food choices and capacity to adhere to a healthier diet or take advantage of opportunities for physical activity (Pampel et al., 2010). Experiencing financial difficulties can expose to stress that directly affects mental health and can impact sense of personal control. Understanding the social determinants of comorbidity can inform public health measures and improve our understanding of the impact of early-life exposures on potentially accumulative health disadvantage.

Sex and/or gender may influence the relationship between social factors and mental and physical health comorbidity. Prior studies of sex differences in the effects of family SEP on BMI (Barriuso et al., 2015; Shrewsbury & Wardle, 2008) or mental health problems (Reiss, 2013; Wirback et al., 2014) have returned inconsistent findings. In general, females report higher levels of emotional problems, and an earlier and higher incidence of depression than males (Kuehner, 2017). At the same time, the relationship between depression and overweight appears to be stronger in females (Mannan et al., 2016; Milaneschi et al., 2019), potentially due to the gendered nature of weight stigma (Puhl & Heuer, 2009).

We examine how parental education, social class and financial difficulties are associated with the comorbidity of depression and overweight in adolescence and early adulthood. These two time-points represent before and after a key life course transition, during which many young people move out of the family home, begin establishing educational qualifications or transition to the labour market, and form important social relationships. We evaluate whether the impacts are modified by age or sex, mapping out the social risk factors for depression-overweight comorbidity. We use rich data from a UK birth cohort, including a validated depressive symptom questionnaire and measured BMI, and prospectively collected measures of socioeconomic indicators.

## 2 Materials and methods

### 2.1 Data

We use data from the Avon Longitudinal Study of Parents and Children (ALSPAC), a population-based prospective birth cohort (Boyd et al., 2013; Fraser et al., 2013; Northstone et al., 2019). ALSPAC recruited 14,541 pregnant women residing in the former county of Avon around the city of Bristol in the South West of England, UK, with an estimated delivery date between April 1991 and December 1992. Of these initial pregnancies, 13,988 children were alive at 1 year of age. When the oldest children were approximately 7 years of age, an attempt was made to bolster the initial sample with eligible cases who had failed to join the study originally. The total sample size for analyses using any data collected after the age of seven is therefore 15,447 pregnancies, of which 14,901 children were alive at 1 year of age. The families have been followed up with regular assessments to the present day. The study website contains details of all the data that is available through a fully searchable data dictionary and variable search tool (http://www.bristol.ac.uk/alspac/researchers/our-data/).

In 2008–2011, at a target age of 17.5 years, 5,217 ALSPAC participants took part in a clinic where weight and height were measured, and 4,500 participants responded to an online questionnaire with data on depressive symptoms. A clinic in 2015-2017 was attended by 4,026 participants at approximately 24 years of age, and 4,222 ALSPAC participants completed a questionnaire including questions on depressive symptoms at approximately 23 years of age. The sample for the present study includes participants who had data on depressive symptoms and/or BMI at the age 17 wave (Figure A.1 in Appendix A). Study data were collected and managed using REDCap electronic data capture tools hosted at the University of Bristol.(Harris et al., 2009). REDCap (Research Electronic Data Capture) is a secure, web-based software platform designed to support data capture for research studies. Ethical approval for the study was obtained from the ALSPAC Ethics and Law Committee and the Local Research Ethics Committees. Informed consent for the use of data was obtained from participants following the recommendations of the ALSPAC Ethics and Law Committee at the time.

### 2.2 Outcomes

As our primary outcomes of interest at target ages 17 and 24, we created multinomial outcome variables of comorbidity categorised as: (i) no depression or overweight (reference), (ii) depression only, (iii) overweight only, and (iv) depression and overweight comorbidity.

BMI (kg/m^2^) was calculated from measured weight and height. At the clinics, height was measured to the nearest millimetre using a Harpenden stadiometer, and weight was measured either to the nearest 0.05 kg with a Tanita Body Fat Analyser (age 17) or to the nearest 0.1 kg using Tanita TBF-401A electronic body composition scales (age 24). For ages under 18, BMI categories of ‘underweight’, ‘normal weight’, ‘overweight’ and ‘obesity’ were identified using sex- and age-specific BMI Z-scores relative to the UK1990 reference population (Vidmar et al., 2013), and at ages 18 and over, by BMI<18.5, BMI≥18.5 and < 25, BMI≥25 and < 30, and BMI≥30 respectively.

To identify possible depression, we used scores from the short Moods and Feelings Questionnaire (sMFQ), a 13-item self-reported questionnaire used in research for screening depressive symptoms in adolescents (Angold et al., 1996), administered through online or paper questionnaires. Participants were asked to rate statements about experiences of low mood and other correlates in the past 2 weeks as “not true”, “sometimes” or “true”. Total scores range between 0 and 26, with higher scores indicating more depressive symptoms. A score threshold of ≥11 was used to indicate depression (Kwong, 2019; Turner et al., 2014).

### 2.3 Exposures

#### Early-life socioeconomic circumstances

Socioeconomic circumstances in early childhood were measured with three indicators: parental education, social class and financial difficulties. Parental education was identified as the highest educational level from the mother’s and her partner’s self-reported qualifications in questionnaires during pregnancy, and categorised as (i) degree-level qualifications, (ii) Advanced (A)-level (examinations around age 18), and (iii) Ordinary (O)-level (examinations taking place at approx. age 16, the UK minimum school leaving age when the mothers were at school), CSE (Certificate of Secondary Education), vocational degree or no qualifications. Parental social class was derived from questionnaires during pregnancy and measured by the highest occupational class based on Registrar General’s Social Class classification: (i) I – professional, (ii) II – managerial/technical, and (ii) III – skilled manual or nonmanual, and (iv) IV – semiskilled manual or V – unskilled manual. Financial difficulties in affording food, heating, or rent or mortgage were assessed in a questionnaire at 32 weeks' gestation. For each, mothers were asked “How difficult at the moment do you find it to afford these items” with the options “Very” “Fairly” “Slightly” or “Not difficult.” Table A.1 details the distribution of the responses to these questions in the sample. We constructed a multinomial categorical measure identifying if there were: (i) no difficulties reported, (ii) any reported as “slightly difficult” but not “fairly” or “very”, (iii) any reported as “fairly difficult” but not “very” and (iv) if any were reported as “very difficult.”

### 2.4 Covariates

We included ethnicity reported by the mother (dichotomized as white/black or minority ethnicity) as a covariate to distinguish the effects of ethnicity from socioeconomic effects. In additional models, we adjusted for maternal depressive score and maternal BMI in early pregnancy as potential confounders, although they may mediate some of the effects of family SEP, as they are likely to be on the causal pathway between family SEP and adolescent health. Maternal depressive score was derived from a version of the Edinburgh Postnatal Depression Scale (EPDS) questionnaire at 32 weeks gestation (median 6.0, interquartile range 3.0-10.0). Maternal BMI was derived from self-reported pre-pregnancy weight and height in a questionnaire during or right after pregnancy (median BMI 22.1, interquartile range 20.5-24.2).

### 2.5 Dealing with missing data

Of the participants who had at least one outcome measure at age 17 (N = 4,948), 66% had any missing data in one or more of the exposures, outcomes or covariates. This varied from none for sex, to less than 3% for education or class, 6% for financial difficulties and 45% for depression at age 24 (Table A.2. After examining patterns of missingness, we used multiple imputation (MI) to increase power and reduce selection bias under a missing-at-random (MAR) assumption. For imputation models, we included depression and BMI, all exposures and covariates from the analytical models, and additional variables that may be predictive of missingness or predict missing values themselves (Table A.2). We imputed data for males and females separately to enable comparisons across sexes. Comorbidity was passively imputed. We generated 50 imputed datasets with 20 cycles and combined coefficients across datasets using Rubin’s rules. MI was performed using the ‘mi impute chained’ command in Stata 17.

### 2.6 Statistical methods

Analyses were conducted with Stata 17.0. First, we examined the sex-specific distributions of depression, BMI categories and depression-overweight comorbidity, and then examined the association between BMI category and depression with logistic regression models separately in girls and boys to better characterize the potential sex-specific association between BMI and depression. We estimated odds ratios (ORs) for the associations between socioeconomic circumstances and depression or overweight with logistic regression. In the main analysis, we modelled depression-overweight comorbidity using multinomial logistic regression models which return relative risk ratios (RRRs). We adjusted for ethnicity and ran the models separately by sex and age and included each exposure (parental education, social class and financial difficulties) separately, as we wished to contrast the three socioeconomic indicators rather than to estimate the direct contribution of each independently of the other indicators. We tested the interactions between socioeconomic measures and sex in each model with joint tests of interaction terms, applying a threshold of p<0.05 for statistical evidence of interaction. We also used the user-written Stata command ‘riigen’ to construct relative indices of inequality (RIIs) for each socioeconomic measure to enable more direct comparison. This approach converts a categorical SEP exposure into a continuous variable ranging between 0 and 1, representing the proportion of the sample with a higher social position, assigning the cumulative percentage point of the median rank within a category to each observation (Mackenbach & Kunst, 1997). The RII therefore compares the hypothetical top and bottom of a continuous SEP distribution. In an additional analysis, we mutually adjusted for the three different socioeconomic RIIs. In sensitivity analyses, we adjusted for maternal depressive score and BMI. We also repeated the main analyses (a) using BMI≥25 as the definition of overweight at age 17 for all, (b) using depression-obesity comorbidity as the outcome, (c) in the unimputed data, and (d) in the unimputed data excluding participants with underweight.

## 3 Results

### 3.1 Main results

The characteristics of the unimputed and imputed data were highly similar (Table 1), and we report the main results using imputed data. Females were more likely to have depression than males regardless of having overweight. Overall, 25.8% of females and 16.5% of males overall had depression at age 17, and of those with overweight, 31.3% had depression in females, and 13.7% in males. Proportions with overweight or obesity at age 17 were similar by sex, while 7.2% of girls and 2.9% of boys had depression and overweight comorbidity. The prevalence of overweight and obesity increased substantially from age 17 to 24, and depression and comorbidity prevalence increased as well. At age 24, 12.6% of females and 8.2% of males had comorbidity. Having overweight and particularly obesity was associated with greater odds of depression in females, while in males the estimates were imprecise with no clear relationship (Table 2).

**Table 1:** Characteristics of the unimputed and imputed sample (N = 4,948).

	Unimputed	Imputed		
	All	All	Females	Males
**Sex, % (N)**				
Male	43.5 (2150)	43.5		
Female	56.5 (2798)	56.5		
**Ethnicity, % (N)**				
White	95.7 (4557)	95.6	95.5	95.6
Other	4.3 (206)	4.4	4.4	4.4
**Depression at 17, % (N)**				
No	78.5 (3332)	78.2	74.2	83.5
Yes	21.5 (913)	21.8	25.8	16.5
**BMI category at 17, % (N)**				
Underweight	7.9 (373)	7.9	7.8	8
Normal weight	69.3 (3279)	69.2	68.2	70.4
Overweight	16.2 (766)	16.3	16.7	15.8
Obesity	6.6 (313)	6.6	7.3	5.7
**Comorbidity at 17, % (N)**				
Has neither depression nor overweight	61.9 (2493)	62.1	59.2	65.9
Depression only	15.9 (640)	15.6	17.7	13
Overweight only	16.9 (682)	16.9	15.9	18.2
Comorbidity	5.3 (213)	5.3	7.2	2.9
**Depression at 23, % (N)**				
No	76.8 (2096)	76.5	73.4	80.5
Yes	23.2 (632)	23.5	26.6	19.5
**BMI category at 24, % (N)**				
Underweight	3.0 (89)	3.9	4.4	3.2
Normal weight	59.6 (1754)	55.8	55.9	55.5
Overweight	24.5 (721)	27.1	24.4	30.5
Obesity	6.6 (313)	13.3	15.3	10.8
**Comorbidity at 24, % (N)**				
Has neither depression nor overweight	50.6 (1108)	46.7	46.4	47.4
Depression only	12.9 (282)	12.8	13.9	11.3
Overweight only	26.9 (588)	30	27.1	33.1
Comorbidity	9.6 (211)	10.7	12.6	8.2
**Highest parental education, % (N)**				
Degree	29.3 (1423)	29.2	27.8	31.1
A-level	35.5 (1721)	35.4	35.2	35.6
O-level/CSE/vocational/none	35.2 (1707)	35.4	37	33.3
**Highest parental social class, % (N)**				
I	13.6 (653)	13.5	12.6	14.7
II	45.0 (2166)	44.9	44.6	45.2
III	37.4 (1802)	37.6	38.8	36
IV/V	4.0 (191)	4.1	4.1	4.1
**Financial difficulties, % (N)**				
No difficulties	66.2 (3079)	65.8	65.1	66.7
Some difficulties	20.8 (967)	20.9	21.3	20.3
Fairly difficult	8.6 (401)	8.8	9	8.4
Very difficult	4.4 (204)	4.5	4.6	4.4
**Maternal EPDS score, mean (SD)**	6.55 (4.84)	6.58	6.63	6.52
**Maternal BMI, mean (SD)**	22.84 (3.70)	22.87	22.82	22.92

**Table 2:** Association between BMI category and depression at ages 17 and 24.

	Females		Males	
Age 17	OR	95% CI	OR	95% CI
Underweight	1.39	1.00, 1.92	1.17	0.76, 1.82
Normal weight	ref		ref	
Overweight	1.31	1.02, 1.69	0.79	0.54, 1.16
Obesity	1.95	1.38, 2.75	0.79	0.43, 1.43
**Age 24**				
Underweight	1.01	0.60, 1.70	0.94	0.38, 2.35
Normal weight	ref		ref	
Overweight	1.37	1.06, 1.78	0.97	0.67, 1.40
Obesity	1.88	1.38, 2.57	1.24	0.74, 2.08

Note: Models adjusted for ethnicity. BMI = body mass index, OR = odds ratio, CI = confidence interval, ref = reference category.

Low parental education, class and greater financial difficulties were all associated with greater odds of depression in females (Table A.3). In males, results for education and class were similar to those seen with girls, but results with financial difficulties were weaker. All three measures were associated with the odds of overweight at age 17 in females (Table A.4), and particularly the lowest parental social class (IV/V) demonstrated a strong association with having overweight. There was less evidence of an association between class or financial difficulties with overweight in males.

**Fig. 1:**
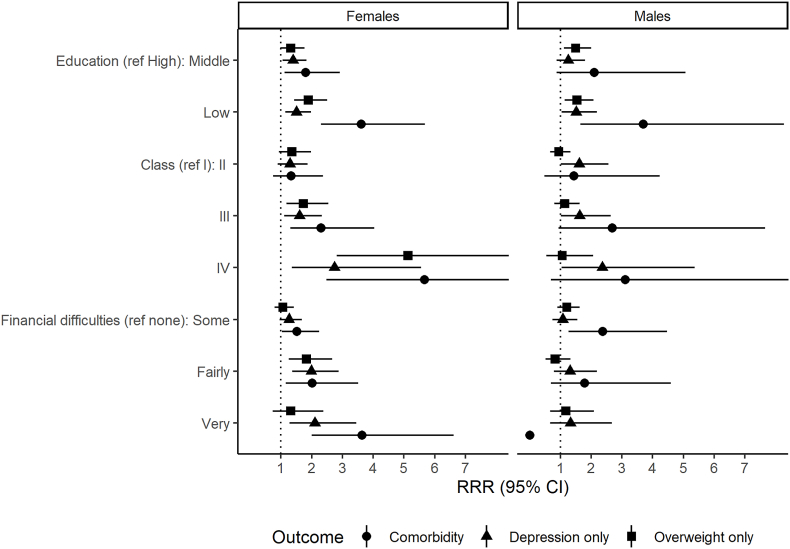
Association between family socioeconomic circumstances and depression-overweight comorbidity at age 17 by sex. Note: Relative risk ratios (RRR) from multinomial logistic regression models of comorbidity versus neither depression or overweight. Models adjusted for ethnicity. Model for comorbidity and financial difficulties in males did not converge due to low numbers. Ref = reference category.

Fig. 1 demonstrates associations of the three measures of socioeconomic circumstances with comorbidity at age 17. Estimates of the associations with comorbidity were greater than those for ‘depression only’ or ‘overweight only’ (Fig. 1, Table A.5). Low education and social class were associated with risk of comorbidity in both sexes. Financial difficulties were a risk factor for comorbidity in females, but the pattern was less clear in males. Testing the evidence for differential SEP effects by sex, we found no statistically significant differences but interaction terms were imprecise (Table A.6), but we report the results by sex due to the differential association of depression and overweight by sex. Education was associated with the highest RII for comorbidity of the three measures in girls and boys, but the confidence intervals were wide and overlapping (Table A.7).

**Fig. 2:**
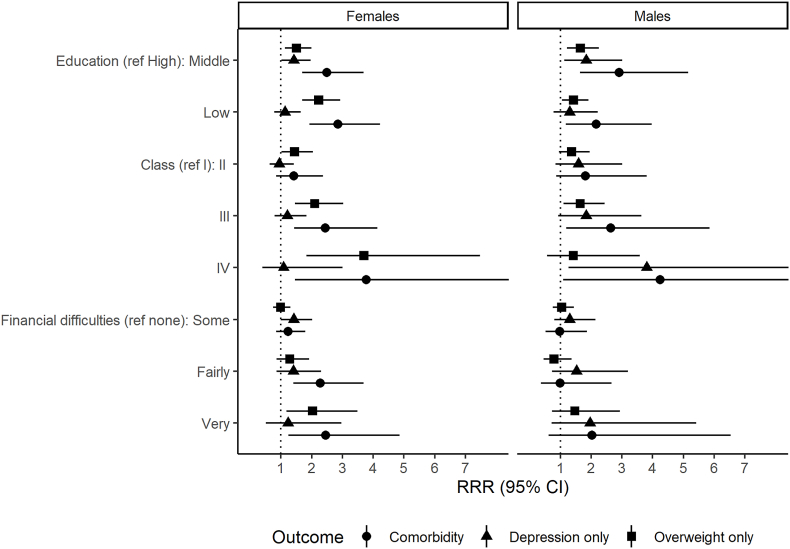
Association between family socioeconomic circumstances and depression-overweight comorbidity at age 24 by sex. Note: Relative risk ratios (RRR) from multinomial logistic regression models of comorbidity versus neither depression or overweight. Models adjusted for ethnicity. Ref = reference category.

At age 24 (Fig. 2, Table A.3, A.4, A.5) , the results for depression, overweight and comorbidity were very similar to age 17. Education had the highest RII for comorbidity for females at age 24, but the RII for class was slightly higher for males (Table A.7). When mutually adjusted, education had the highest RII for girls at both age 17 and 24 and for boys at age 24, but again the confidence intervals were wide and overlapping (Table A.8).

### 3.2 Sensitivity analyses

Results additionally adjusting for maternal depressive symptoms and BMI attenuated the RRR for comorbidity by between 22 and 42% for education, 27 and 55% for class, and 13 and 48% for financial difficulties at age 17, but did not remove them (Table A.9). Using BMI≥25 definition of overweight at age 17 showed similar results (Table A.10). Defining comorbidity with obesity rather than overweight generally showed slightly greater risk associated with socioeconomic measures, but confidence intervals were wider (Table A.11). Finally, results for the main models in unimputed data were highly similar to the imputed data (Table A.12) and when excluding participants with underweight (Table A.13).

## 4 Discussion

Using data of adolescents coming of age in UK in the 2000s, we found that early-life parental socioeconomic circumstances were associated with depression, overweight and their comorbidity in adolescence and young adulthood. Parental education, social class and financial difficulties were all risk factors for comorbidity, and generally more strongly for comorbidity than depression only or overweight only. The findings were similar from adolescence to young adulthood, and we did not find strong evidence of sex differences.

The comorbidity of obesity and depression is likely to be one of the earliest life course manifestations of multimorbidity, with potentially serious lifelong implications for subsequent chronic diseases and wellbeing. The findings contribute to our understanding of the early social causes by comparing relationships of different socioeconomic risk factors. Our results corroborate previous findings of an association between parental social class and comorbidity (Khanolkar & Patalay, 2021). However, our results indicate that parental education tended to be the most consistent indicator of comorbidity risk and in most cases had the highest RII estimates, indicating a greater total population impact. In additional models we mutually adjusted for the three SEP RIIs, and the RII for education tended to be also the highest in these models, but the meaning for the resulting estimates are likely to vary: for education we are adjusting for mediators, and for class and financial difficulties we may be adjusting for a mix of confounders and mediators. Parental education influences several aspects of socioeconomic circumstances such as occupation, income, financial difficulties, housing and neighbourhood, but may also be more strongly associated with physical activity and diet, which are likely to have an impact on depression-overweight comorbidity. Mirowsky and Ross suggested that self-efficacy plays a part in the effects of education on health (Mirowsky & Ross, 2003). More educated parents may have the resources to apply a greater emphasis on fostering health promoting behaviours in the family environment.

The relationships between social class or financial difficulties and the outcome indicate the impact of early-life material conditions. We did not have a measure of family income, but self-reported difficulties in affording food, heating and accommodation capture significant aspects of poverty. Experiencing poverty can be a substantial source of stress that may influence parenting and contribute to household conflict (Conger et al., 1992), and reduce emotional coping resources as well as material resources for healthy life styles. We used a measure of financial difficulties from early-life, but it is possible that financial difficulties experienced in adolescence would have a greater impact on concurrent mental and physical health problems and should be explored further. Irrespective, an increase in families with children experiencing financial difficulties is likely to have significant short and long term effects on adolescent health and wellbeing. Furthermore, the conceptualization and measurement of financial difficulties in our study may impact the estimates, and with a more detailed measure we may have detected more fine-grained effects.

We presented the results by sex, because females show a stronger association between depression and overweight and greater prevalence of comorbidity. These results indicate patterns and processes influenced by sex and/or gender, such as experiences of weight stigma and body satisfaction (Puhl & Heuer, 2009). However, we did not find statistical evidence for sex differences in the impact of SEP, which is in agreement with a previous study using earlier British cohorts examining father’s social class and comorbidity in childhood (Khanolkar & Patalay, 2021).

An advantage of the study was its prospective study design, with socioeconomic measures reported in early-life by mothers and the follow up of the same cohort through adolescence and young adulthood. BMI was measured by trained staff, and the measure of depression, although not used to measure clinical depression, is likely to be more sensitive and less influenced by socioeconomic background than relying on a clinical diagnosis of depression from primary care data which may be influenced by the likelihood of seeking care. However, one of the limitations of the study was the potential bias and low precision in the results due to study attrition and missingness on individual items. Loss to follow-up in ALSPAC is not random (Cornish et al., 2021) and those with poorer health and lower SEP are more likely to drop out, which can bias estimates of SEP differences in health towards the null (Howe et al., 2013). We mitigated this problem by using the maximum sample available, i.e. those with any outcome data at age 17, and applying MI for missing data. In terms of the outcomes, the imputation had little impact on the prevalence of depression, but increased the prevalence of overweight/obesity at age 24. This prevalence is comparable to national reference data (Health Survey for England 2017, 2018). Generally, we also found that results in the unimputed and imputed data were similar. Nevertheless, ALSPAC remains a more advantaged sample than the general English population at the time and predominantly white (Fraser et al., 2013), which may have an impact on generalizability of the findings. For example, the socioeconomic patterning of childhood obesity has been found to vary by ethnic group in the UK (Goisis et al., 2019).

### 4.1 Conclusions

In conclusion, socioeconomic circumstances in early-life predict depression and overweight comorbidity in adolescence and young adulthood, highlighting the early emergence of socioeconomic inequalities in multimorbidity. Future research should ascertain how family SEP is associated with the longitudinal reciprocal relationship between BMI and depressive symptoms in adolescence.

## Financial disclosure

LDH declares grants from the 10.13039/501100000265Medical Research Council and Health Foundation and membership of Academy of Finland funding panel.

## Funding

This work was supported by the 10.13039/501100000269Economic and Social Research Council (grant ES/T013923/1). The UK 10.13039/501100000265Medical Research Council and 10.13039/100004440Wellcome (grant: 217065/Z/19/Z) and the 10.13039/501100000883University of Bristol provide core support for ALSPAC. A comprehensive list of grants funding is available on the ALSPAC website (http://www.bristol.ac.uk/alspac/external/documents/grant-acknowledgements.pdf); The collection of some of the variables used in this study were specifically funded by 10.13039/100010269Wellcome Trust and 10.13039/501100000265MRC (076467/Z/05/Z and MR/L022206/1). This publication is the work of the authors and FK will serve as guarantor for the contents of this paper. The funders had no role in study design, analysis or interpretation of data or decision to submit the article for publication.

## Data access statement

The informed consent obtained from ALSPAC participants does not allow the data to be made freely available through any third party maintained public repository. However, data used for this submission can be made available on request to the ALSPAC Executive. The ALSPAC data management plan describes in detail the policy regarding data sharing, which is through a system of managed open access. Full instructions for applying for data access can be found here: http://www.bristol.ac.uk/alspac/researchers/access/. The ALSPAC study website contains details of all the data that are available (http://www.bristol.ac.uk/alspac/researchers/our-data/).

## Ethical statement

Ethical approval for the study was obtained from the ALSPAC Ethics and Law Committee and the Local Research Ethics Committees. Informed consent for the use of data was obtained from participants following the recommendations of the ALSPAC Ethics and Law Committee at the time.

## CRediT authorship contribution statement

**Fanny Kilpi:** Conceptualization, Formal analysis, Funding acquisition, Investigation, Methodology, Writing – original draft, Writing – review & editing. **Laura D. Howe:** Investigation, Methodology, Writing – review & editing.

## Declaration of competing interest

FK declares no competing interests. LDH declares grants from the 10.13039/501100000265Medical Research Council and Health Foundation and membership of Academy of Finland funding panel.

## Data Availability

Data used for this submission can be made available on request to the ALSPAC Executive. Instructions for applying for access can be found here: http://www.bristol.ac.uk/alspac/researchers/access/.
